# Histomorphological changes in autologous anterior cruciate ligament graft failure show predominantly inflammatory reactions, fissure defects and osteonecrosis

**DOI:** 10.1002/jeo2.70545

**Published:** 2025-11-28

**Authors:** Steffen F. Siemoneit, Alexander Bosse, Steffen T. Ubl, Daniel Günther, Julius M. Wehrmann, Bertil Bouillon, Thomas R. Pfeiffer

**Affiliations:** ^1^ Department of Orthopaedic Surgery, Trauma Surgery and Sports Medicine, Cologne Merheim Medical Center Witten/Herdecke University Cologne Germany; ^2^ Department of Pathology, Klinikum Stuttgart University of Tübingen Stuttgart Germany

**Keywords:** ACL, foreign body reactions, histology, knee, regression phenomena, revision

## Abstract

**Purpose:**

The aim of this study was to identify morphological alterations that occur in graft failure following anterior cruciate ligament (ACL) reconstruction. It was hypothesised that multiple morphological regression phenomena are present at the time of revision surgery.

**Methods:**

In this prospective study, 43 consecutive patients undergoing revision surgery due to graft failure after autologous ACL reconstruction were analysed. Specimens were redrilled with a hollow reamer to obtain the entire drill channel and ACL remnants, which were examined histopathologically using haematoxylin and eosin (HE), Masson‐Goldner, Elastika‐Gieson and CD68+ stains to characterise ligamentous and osseous changes qualitatively and quantitatively under light microscopy.

**Results:**

A total of 76 bone blocks were obtained from 43 patients (mean age 29.67 ± 9.02 years; range 18–58). The cohort included 9 women (20.9%) and 34 men (79.1%). The mean time from graft failure to revision surgery was 53.4 months (range 0.23–84). Femoral fixation in the primary surgery used extracortical button fixation in 87.5% and the All‐Press‐Fit technique in 12.5%, while tibial fixation most commonly involved interference screws (86.4%). Spontaneous trauma was rare (2.5%), with inadequate trauma in 7.5%; 90% of failures followed adequate trauma. Histomorphologic analysis revealed various influential regression phenomena, predominantly central within the autograft. Findings included chronic histiocytic inflammation with associated capillary proliferation, fissure defects and osteonecrosis. Residual suture material emerged as a potential pathological factor contributing to severe regression and destructive changes. Occasionally, degenerative cysts, chondral metaplasia, myxoid degeneration, osteoporotic changes and ectopic ossifications were observed.

**Conclusion:**

Several histomorphological changes have been identified, each with the potential to contribute to graft failure following ACL reconstruction. The most prevalent were fissure defects, inflammatory reactions, osteonecrosis, capillary proliferation and foreign body reactions. Less common are myxoid degenerations, cystic alterations, ectopic ossifications, chondral metaplasia and osteoporotic changes. Their collective impact on graft stability and long‐term clinical outcomes warrants further investigation.

**Level of Evidence:**

Level III, cohort study.

**Abbreviations**
ACL: anterior cruciate ligament
CD68+: cluster of differentiation 68+
EDTA: ethylenediaminetetraacetic acid
FFPE: formalin‐fixed paraffin‐embedded
HE: haematoxylin and eosin

## INTRODUCTION

The incidence of graft failure in anterior cruciate ligament (ACL) surgery has been documented to be as high as 25%, in male patients even higher than in females, constituting a persistent challenge in surgical practice [4, 10, 17]. The MARS Group has identified potential causative factors. The analysis of the primary causes revealed that in 32% of cases, the cause was attributed to traumatic factors, while in 24% of cases, technical errors were identified, particularly due to the misplacement of the femoral drill channel. A further 7% of cases were attributed to biological failure and 37% to a combination of the mentioned factors. The proportion of cases resulting from infection was found to be less than 1% [21]. The term ‘biological failure′ describes instances in which there are no identifiable surgical, biomechanical or traumatic causes for the graft failure. In contrast to an obviously misplaced drill channel, a biological failure is difficult to visualise as the processes take place at a cellular level. Despite the fact that the use of autografts has emerged as the preferred therapeutic approach following a combination of surgical, clinical, functional and morphological assessments, the aetiology of graft failure is complex and involves a number of histopathological processes that remain poorly understood. The regression phenomenon is a general term for the different histomorphological changes. The current literature is predominantly derived from animal experiments or individual case studies. Histopathological examinations of primary injuries to the Achilles tendon, the rotator cuff and the quadriceps tendon provide parallels to the results described below. In this context, pictures of fissural splitting, infiltration of inflammatory cells, capillary proliferation, myxoid degeneration, chondral metaplasia and ossifications have been described before. Nevertheless, there have been no specific studies on graft failure after ACL reconstructive surgery [5, 7, 11, 20].

The objective of this study was to describe and characterise the morphological regression phenomena in the ligamentous and osseous compartments at the time of ACL revision surgery. It has been hypothesised that multiple regression phenomena are present at the time of ACL revision surgery following ACL graft failure.

## MATERIALS AND METHODS

### Study population and examination material

This prospective study was approved by the Institutional Review Board and ethics committee of the University of Tuebingen. The cohort was assessed for age, gender, as well as chronic cardiovascular and renal diseases, smoking, metabolic disorders, thrombosis, allergies and tumours. Inclusion criteria were as follows: (1) Primary ACL graft failure, regardless of its cause; (2) single ligament injury; (3) specimens obtained from femoral and tibial drill holes and from the intra‐articular ACL remnant; (4) sufficient quantity of tissue for staining; (5) Hamstring autografts. Samples with inadequate amounts of material, patients with chronic diseases, thrombosis or allergies were excluded.

**Figure 1:**
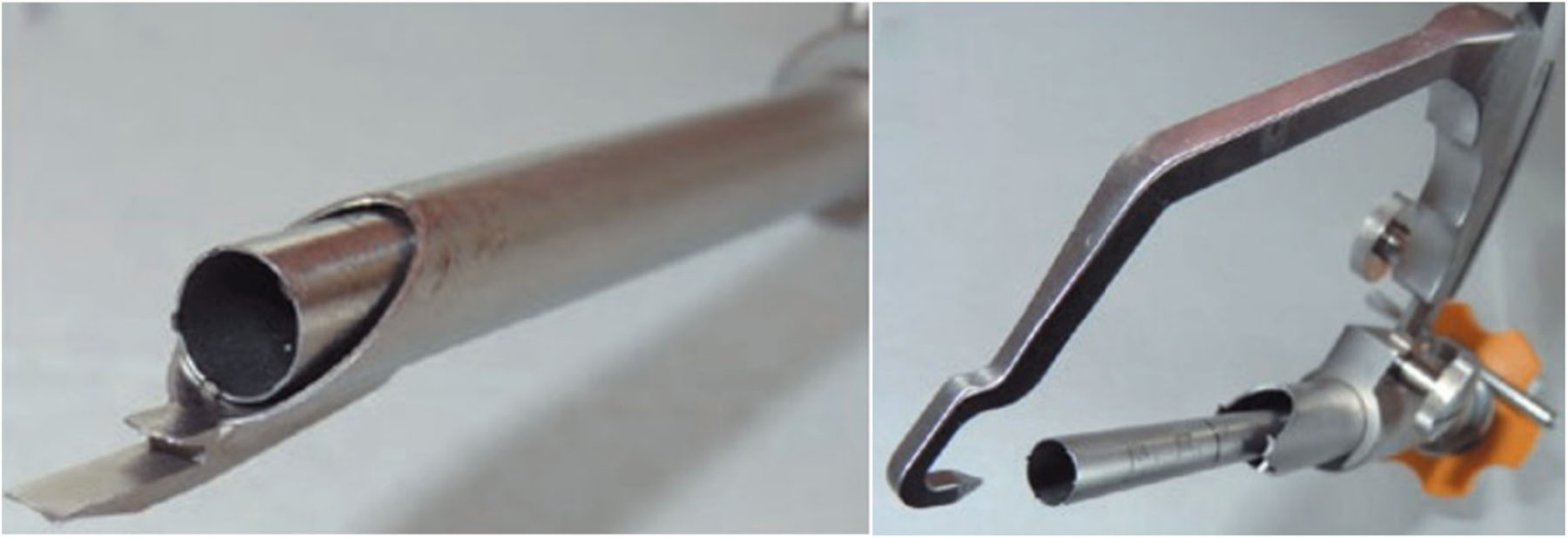
Hollow reamer, femoral (left) and tibial guide (right).

During arthroscopic ACL revision surgery, a total of 76 samples were harvested by drilling over the old tibial and femoral bony drill tunnels with a hollow reamer (diameter 10 ± 2 mm) (Figure 1) in the exact insertion area of the failed autograft to gain the periarticular (near‐joint) portion autograft covered by spongy or cortical bone.

**Figure 2:**
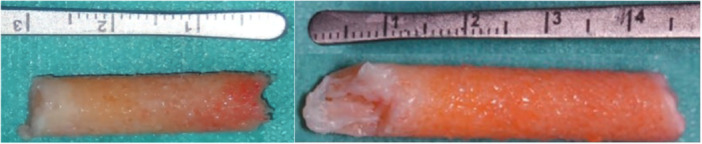
Nine‐mm diameter bone cylinder femoral (left) tibial (right).

The bone cylinder is removed inside the hollow reamer (Figure 2). Subsequently, the drill channels were filled with bone material. The original specimens were fixed in 4% formalin solution and subsequently sent to the local Institute of Pathology for histological examination.

### Specimen preparation

The 4% formalin‐fixed paraffin‐embedded (FFPE) specimens were prepared according to a defined protocol. Bone blocks were first decalcified with ethylenediaminetetraacetic acid (EDTA) and then embedded in paraffin. Subsequently, 2 µm thick sections were cut with a microtome and stained microscopically with HE, Masson‐Goldner, Elastika‐Gieson and CD 68+ stains. Finally, the preparations were analysed and evaluated by light microscopy (Zeiss 2.5×/5×/10×/20×/40× eyepieces at a tube factor of 1.25).

### Outcome measurements

Following light microscopic assessment, specimens were analysed for the histomorphologies: inflammation, fissural splitting, osteonecrosis, capillary proliferation, foreign material, cysts, myxoid degeneration, chondral metaplasia, ectopic ossifications and osteoporotic alterations (Table 1). In addition, the localisation of the detected regression phenomena was investigated. The severity of each observation was graded using a study‐specific grading method for each field of view. This grading method was qualitative and semiqualitative in nature, and the severity of each observation was assessed on a four‐point scale: 0 = no finding, 1 = mild, 2 = moderate, 3 = severe. Specimens were categorised as femur, tibia and ACL remnant (ACL remnant group *n* = 7; separate analysis not performed). Assessments were performed by a medical student with histomorphology expertise and a professor of pathology; discrepancies were resolved by consensus. Descriptive statistics (mean, standard deviation for metric parameters; proportion of findings) were calculated using SPSS 26.0 (IBM). Values reflect the means of the absolute counts for femur and tibia. An AI‐supported programme was employed to rectify grammatical errors.

**Table 1:** Description of histolomorphological changes in autologous tendon grafts and bone tunnels.

Inflammation	Presence of histiocytic inflammatory cells infiltrating the graft or surrounding bone tunnel
Fissural splitting	Loosening and longitudinal splitting of collagen fibres within the tendon graft
Osteonecrosis	Small, nonvital bone trabeculae lacking osteocyte nuclei, indicating avascular bone necrosis
Capillary proliferation	Neoangiogenesis with dense capillary networks, often seen as part of chronic inflammatory response.
Foreign material	Residual synthetic material visible under polarised light due to birefringence
Cysts	Large, round cystic spaces or tendinolyses within the autologous tendon graft
Myxoid degeneration	Replacement of normal tendon matrix by mucinous tissue
Chondral metaplasia	Presence of cartilage‐like cells, embedded in chondroid matrix, within the tendon tissue
Ectopic ossifications	New bone formation within the tendon, sometimes with central fatty marrow
Osteoporotic changes	Loss of transverse and longitudinal trabecular connections, indicating reduced bone density

## RESULTS

### Patient collection

**Figure 3:**
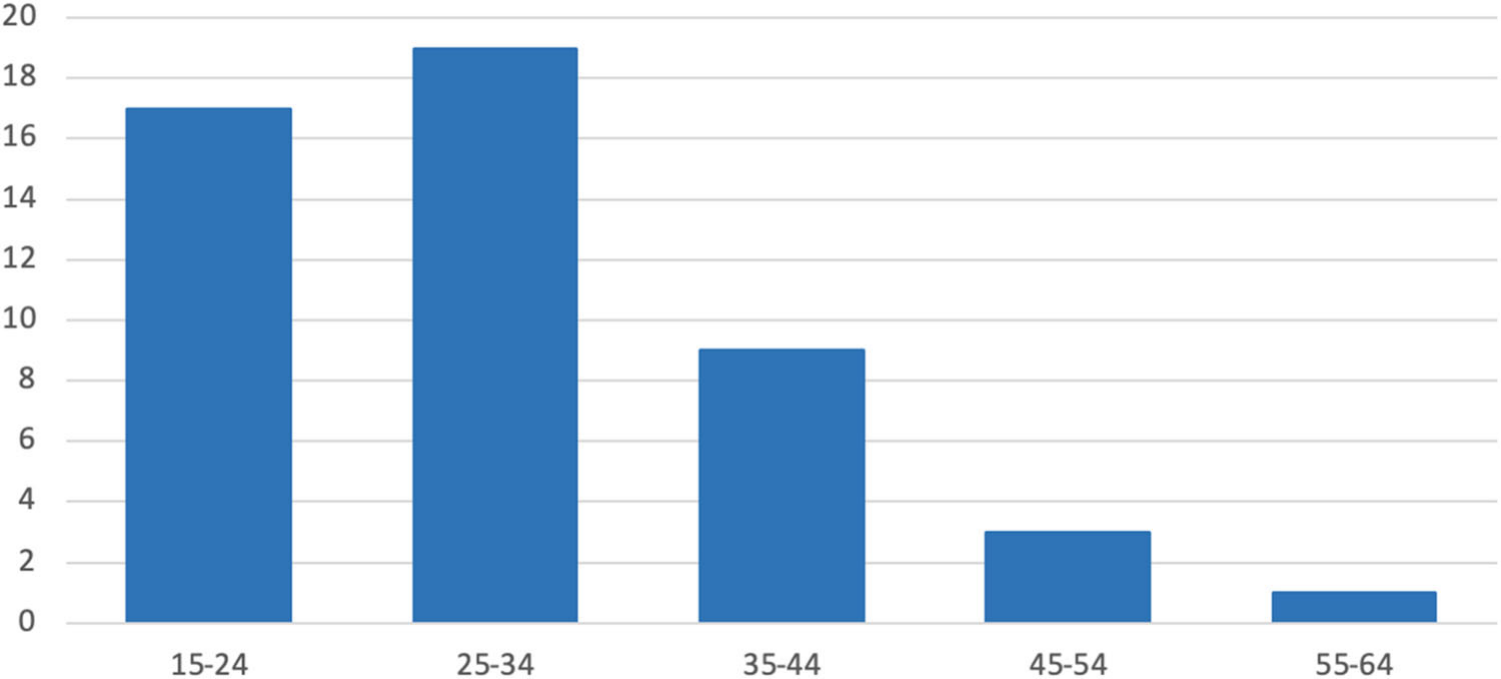
Age (years) distribution by time of revision surgery.

The patient cohort comprised 43 individuals (9 female and 34 male) who underwent revision surgery of the ACL between 2017 and 2019. The age range was 18 to 58 years, with a mean of 29.67 years and a standard deviation of 9.02 years (Figure 3). The gender distribution included nine women (20.9%) and 34 men (79.1%). All patients were in good health and did not have any long‐term illnesses, thrombosis, allergies or cancer. Seventeen of the patients had a history of smoking. With regard to the surgical technique employed in the primary operation different techniques were used for femoral and tibial fixation (Table 2). All used autografts were hamstrings.

**Table 2:** Distribution of the femoral and tibial fixation techniques used in the primary operation.

	Extraarticular button	Interference screw	Suture disk	All press fit
Femoral	28 (65.1%)	4 (9.3%)	0	11 (25.6%)
Tibial	2 (6.1%)	19 (57.6%)	1 (3%)	11 (33.3%)

Regarding the interval between primary and revision surgery, the shortest period was 5.36 months, while the longest was 18.1 years. Spontaneous trauma occurred only once (2.5%) in the study series, while inadequate trauma (i.e., when running) occurred in 7.5%. Ninety percent of all re‐ruptures were due to adequate trauma (pivot sports).

### Tissue sampling

The analysis yielded 30 samples resulting from the femoral drill canals, 39 from the tibial drill canals, and seven were intra‐articular ACL remnants. The variation in distribution can be attributed to the exclusion of material. This is particularly evident in the ACL remnants. The femoral cancellous bone cylinder ranged in size from 10 × 5 × 5 to 35 × 12 × 8 mm in length, width and height. The tibial bone cylinders had a size range of 15 × 5 × 3 to 45 × 12 × 8 mm. The analysis of graft failures revealed a wide range of regression phenomena, varying in quality and quantity (Tables 3 and 4). In total, 10 recurrent regression phenomena were identified in the course of analysis.

**Table 3:** Qualitative analysis of common regression phenomena.

	No findings	Mild	Moderate	Severe
Inflammation	2 (2.8%)	31 (47.9%)	22 (35.2%)	8 (14.0%)
Femoral	1 (3.8%)	13 (50%)	10 (38.5%)	2 (7.7%)
Tibial	1 (2.7%)	18 (48.6%)	12 32.4%	6 (16.2%)
Fissural splitting	0	17 (26.8%)	30 (49.3%)	16 (23.9%)
Femoral	0	8 (27.6%)	15 (51.7%)	6 (20.7%)
Tibial	0	9 (26.5%)	15 (44.1%)	10 (29.4%)
Osteonecrosis	1 (6.0%)	30 (52.2%)	21 (31.3%)	7 (10.4%)
Femoral	1 (3.8%)	12 (46.2%)	9 (34.6%)	4 (15.4%)
Tibial	0	18 (50%)	12 (33.3%)	3 (8.3%)
Capillary proliferation	0	31 (47.9%)	25 (38.0%)	7 (14.1%)
Femoral	0	15 (57.7%)	9 (34.6%)	2 (7.7%)
Tibial	0	16 (43.2%)	16 (43.2%)	5 (13.5%)
Foreign material	4 (7.3%)	33 (55.9%)	11 (19.1%)	12 (17.6%)
Femoral	2 (8.3%)	13 (54.2%)	4 (16.7%)	5 (20.8%)
Tibial	2 (5.6%)	20 (55.6%)	7 (19.4%)	7 (19.4%)

**Table 4:** Qualitative analysis of occasional regression phenomena.

	No findings	Mild	Moderate	Severe
Cysts	47 (69.6%)	11 (20.3%)	5 (8.7%)	1 (1.4%)
Femoral	22 (81.5%)	4 (14.8%)	1 (3.7%)	0
Tibial	25 (67.6%)	7 (18.9%)	4 (10.8%)	1 (2.7%)
Myxoid degeneration	36 (53.5%)	20 (32.4%)	5 (11.3%)	2 (2.8%)
Femoral	17 (65.4%)	8 (30.8%)	0	1 (3.8%)
Tibial	19 (51.4%)	12 (32.4%)	5 (13.5%)	1 (2.7%)
Chondral metaplasia	69 (85.7%)	4 (6.0%)	7 (8.3%)	0
Femoral	38 (95%)	0	2 (5%)	0
Tibial	31 (77.5%)	4 (10%)	5 (12.5%)	0
Ectopic ossifications	78.9%	12.7%	5.6%	2.8%
Femoral	85.2%	11.1%	3.7%	0
Tibial	77.8%	11.1%	8.3%	2.8%
Osteoporotic alterations	55 (80.9%)	4 (5.9%)	6 (8.8%)	3 (4.4%)
Femoral	25 (83.3%)	0	4 (13.3%)	1 (3.3%)
Tibial	30 (78.9%)	4 (10.5%)	2 (5.3%)	2 (5.3%)

### Topography

The regression phenomena under examination are located in the central region of the graft, which is located peripherally to the adaptation zone between the autograft and the bone. Even in cases of homogeneous adaptation inside the drill channels, destructive changes in the tendon can frequently be observed in the periphery. The definition of a successful adaptation is characterised by vertically radiating Sharpey fibres and is devoid of any regression phenomena. A homogenous adaption zone was identified in a small percentage of tibial subjects (8.3%), while successful adaption with only mild regressive changes in the periphery was observed in 28% of the femoral specimens and 38.9% of the tibial specimens.

### Common regression phenomena

**Figure 4:**
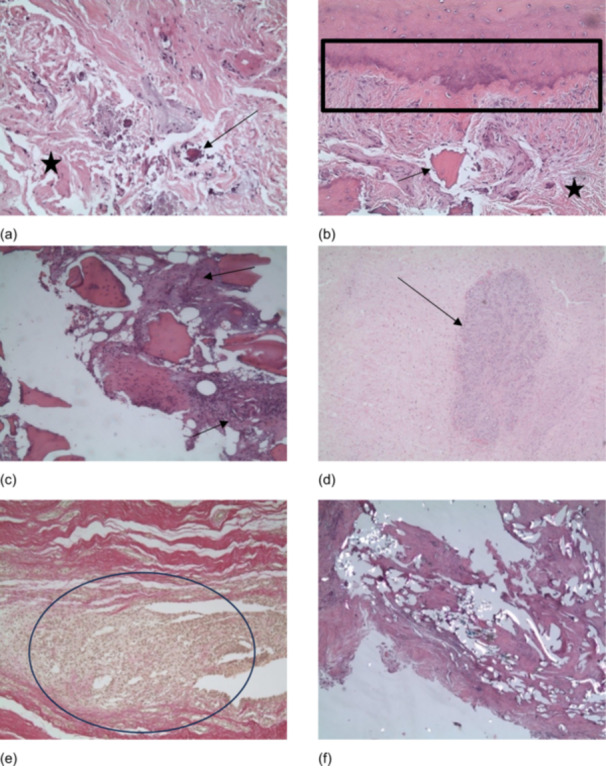
Common regression phenomena. (a) The image displays high‐grade degenerative changes (asterisk) and patchy osteonecrosis (arrow). These are embedded in a completely destroyed tendon with sandwich‐like fissure defects and interspersed with isolated round cells indicating chronic inflammation. haematoxylin and eosin (HE) x10. (b) Correct adaptation (box) is accompanied by notable degenerative alterations in the tendon region, with the involvement of devitalised bone (arrow) and splitting phenomena (asterisk). HE 5x. (c) Chronic histiocytic necrotising inflammation of the bony structures, as indicated by the arrows. The image is analogous to that of sequestering and necrotising osteomyelitis. HE 5x. (d) Nodular capillary proliferation (arrow) with endothelial proliferation, accompanied by a relatively calm tendon structure that displays minimal signs of inflammation. HE 5x. (e) Pronounced nodular granulomatous inflammation with increased histiocyte infiltration (circle). HE 10x. (f) Clear accumulation of birefringent suture remnants with marked destruction of the autograft and bony structures. HE 5x.

The most prevalent findings were osteonecrosis (94.0%), inflammatory reactions (93.0%) and fissure defects (91.7%) (Figure 4a–c), frequently manifesting at the periphery of the adaptation zone, but also extending into the adaptation zone. In 73.2%, fissure defects were at least moderate. In particular, histiocytic changes were the dominant feature of the inflammatory reactions, contributing significantly to the regression of the implanted graft and, in severe cases, of the bone. The presence of corresponding capillary proliferation (91.7%) (Figure 4d) and round cell infiltrates was also observed (Figure 4ac,e).

Individual foreign body reactions (92.6%) (Figure 4f) directly caused the formation of intraligamentous cysts and fissural splitting, representing a residual condition. These images of birefringent suture remnants were directly associated with necrotic alterations and a pronounced inflammatory response. 17.6% of all cases exhibited the most severe form of foreign body reactions.

The different qualitative characteristics of the regression phenomena identified are presented in Table 3 below.

### Occasional regression phenomena

**Figure 5:**
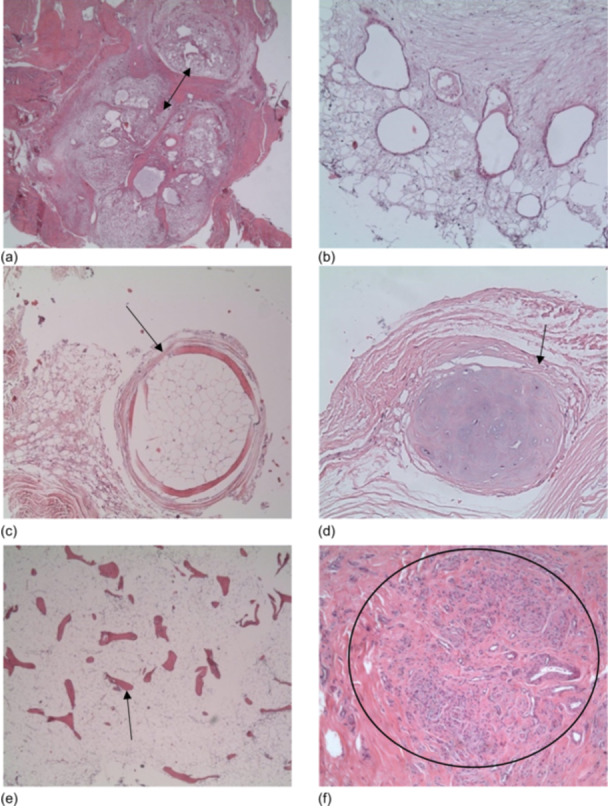
Occasional regression phenomena. (a) Pronounced myxoid degeneration of the tendon structure, interspersed with round cells, fissure defects and cyst formation, with the inclusion of mucous deposits (arrows) haematoxylin and eosin (HE) 5x. (b) Cyst formation with lipomatous metaplasia HE 5x. (c) Ectopic ossification with central fat marrow and scattered haematopoietic cells (arrow). HE 5x. (d) Chondral metaplasia (arrow) within a regressive and fissured tendon structure. HE 5x. (e) Typical osteoporotic remodelling of bone tissue (arrow), which is characterised by a loss of both longitudinal and transverse cross‐linking. HE 2,5x. (f) Scar neuroma (circle), characterised by a proliferation of nerve fibres in a shrub‐like configuration and reactive vascularisation. HE 10x.

**Figure 6:**
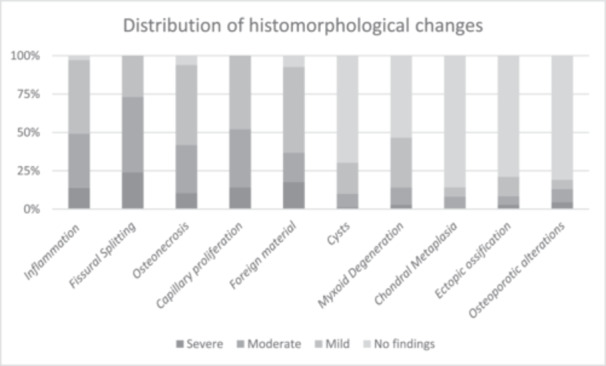
Distribution of histomorphological changes.

In the context of chronic inflammatory reactions, two associated regressive changes were observed in the autograft: myxoid degeneration (43.4%) (Figure 5a) and cystic alterations (30.4%) (Figure 5b). Additionally, recurrent lipomatous atrophy of the ligamentous structures, as well as ectopic ossifications (19.7%) (Figure 5c) and multiple small nodular chondral metaplasia (15.7%) (Figure 5d) were observed. On occasion, osseous structures exhibited clear osteoporotic alterations, characterised by a distinctive rounding of the affected bony areas (17.1%) (Figure 5e). Additionally, a scar neuroma was identified in one specimen (Figure 5f). Table 3 presents the different qualities of the less common regression phenomena. Conclusively, Figure 6 provides a summary of the distribution of the common and occasional histomorphological changes.

## DISCUSSION

The main finding of this study is the occurrence of multiple regression phenomena in both ligamentous and osseous compartments, resulting in histomorphological alterations of autologous tendon grafts and bony drill tunnels. Analyses of graft failures underline the importance of recognising predisposing factors, particularly morphological changes. Early detection and improved understanding of these mechanisms can significantly reduce the risk of failure and enhance revision outcomes and prognosis [13].

### Morphological causes of graft failure

Recent research suggests that nontraumatic causes‐technical, morphological and biological‐represent the primary determinants of graft failure [14, 22]. Histological analyses of failed ACL grafts have shown patterns consistent with our findings, though generally less detailed [2, 14, 16]. The most frequent alterations include osteonecrosis, inflammatory reactions and fissural splitting, which have also been reported in Achilles tendon ruptures [8]. Fissural splitting and inflammation, often interpreted as the final stage of degeneration [7], highlight the parallels between ACL failure and other tendinopathies. Similarly, histological findings in rotator cuff tears demonstrate thinning and disorganisation of collagen fibres, myxoid and hyaline degeneration, chondroid metaplasia, calcifications, vascular proliferation and fatty infiltration, though without inflammatory responses [5]. Thus, morphological changes observed in failed ACL grafts reflect general pathohistological processes rather than unique entities.

Osteonecrosis and fissure defects exert a considerable influence on graft incorporation, often arising at the periphery of the adaptation zone but occasionally extending into it (Figure 4a,b). Possible causes include thermal necrosis from drilling femoral and tibial tunnels or mechanical trauma during graft fixation. Further research is needed to confirm these assumptions. Histiocytic inflammatory reactions, commonly associated with cystic alterations and capillary proliferation (Figure 4c–e), further compromise the remodelling process, preventing organised collagen fibre arrangement [16]. Our findings align with reports of macrophage, giant cell and lymphocyte accumulation in spontaneous graft failures [2]. Excessive inflammation may also contribute to tunnel widening, peri‐tunnel bone loss and graft degeneration [24].

Other morphological changes, such as chondral metaplasia, myxoid degeneration, ectopic ossification and osteoporotic alterations, occurred less frequently (Figure 5a–e). These changes, documented in animal studies, are associated with significantly reduced graft stability [6]. Chondral metaplasia has similarly been described in Achilles tendons exposed to abnormal loading, resulting in reduced tensile resistance [12]. A meta‐analysis demonstrated consistently reduced bone mineralisation after ACL reconstruction [18]. One year postoperatively, bone density in the operated knee remains significantly lower than in the contralateral knee [15], suggesting impaired prerequisites for drill‐channel integration due to insufficient mineralisation.

Myxoid degeneration, widely considered a marker of advanced degeneration [5, 11], was further supported by comparative analyses of native quadriceps and rotator cuff tendons. Ectopic ossification, also identified microscopically in patients with paraplegia, results from altered tension and compression vectors. Similarly, ankylosing ossifications have been observed under conditions of spasticity in the capsuloligamentous apparatus of the pelvis [1]. These phenomena underscore the importance of biomechanical forces in ACL reconstruction. Myositis ossificans traumatica, triggered by traumatic injury, exemplifies the interplay of haemorrhage, inflammation and ectopic ossification [9].

Foreign material residues may further contribute to graft degeneration. Microscopic analysis revealed inflammatory responses, necrosis and cystic changes associated with birefringent suture material (Figure 4f), similar to findings in Achilles tendon ruptures [19]. This suggests that minimising foreign material or exploring alternative fixation strategies could reduce the risk of graft weakening.

### Other factors contributing to regression phenomena?

ACL graft failure results from the interplay of technical, morphological and biological factors. Technical factors include nonanatomical tunnel placement, premature return to sport, overly aggressive rehabilitation, repetitive trauma, prolonged immobilisation, or transplant micromotion. Biological factors encompass infection, arthrofibrosis and synovial fluid effects. Morphological causes involve graft necrosis and impaired ligamentisation [14].

Although the hypothesis of ‘synovial bathing’—the accumulation of fluid in dead spaces between graft and bone—was refuted [3], specific cytokines in synovial fluid may promote morphological alterations [16]. Tunnel widening and graft micromotion, described as ‘stress shielding’, also impair osseous integration. The ‘windshield wiper effect’ in the sagittal plane and the ‘bungee effect’ in the longitudinal plane negatively influence tendon graft healing [23]. Such micromovements compromise graft integrity, manifesting as fissural splitting or inflammatory changes.

From a clinical perspective, the primary objective is to prevent the onset of these histomorphological alterations, as they have the potential to compromise the integrity of an ACL reconstruction. In particular, it is imperative that the underlying causes of this phenomenon are thoroughly investigated within this specific context. It is imperative that further investigation be conducted into the influence of foreign body reactions and heat necrosis.

### Limitations

This study has several limitations. First, the use of human specimens precluded the creation of a control group of intact ACL grafts, which limits the ability to determine whether the morphological changes observed are specific to failed grafts or represent normal adaptations following ACL reconstruction. Second, a study‐specific grading method was used for qualitatively and semi‐qualitatively grading of the histomorphological findings. Third, the inclusion of 43 patients may have limited the power to detect less common morphological changes. Fourth, the time interval between graft failure and revision surgery may have influenced the morphological changes observed, as longer periods could potentially result in more frequent alterations. Finally, although histological examination provides insight into the morphological changes associated with graft failure, it cannot definitively establish causality. The multifactorial cause of ACL graft failure, which includes the aforementioned factors, limits conclusions about the underlying aetiology.

## CONCLUSION

Several histomorphological changes have been identified, all of which have the potential to precipitate graft failure following ACL reconstruction. The most prevalent morphological regression phenomena include fissure defects, inflammatory reactions, osteonecrosis, capillary proliferation and foreign body reactions. Less common are myxoid degenerations, cystic alterations, ectopic ossifications, chondral metaplasia and osteoporotic changes. Their impact on the overall stability of an ACL reconstruction needs to be further investigated.

## AUTHOR CONTRIBUTIONS

Steffen F. Siemoneit, Thomas R. Pfeiffer and Alexander Bosse were involved in the design of the study, data collection and carrying out the histomorphological investigations as well as quantitative measurements. The complete article was written by Steffen F. Siemoneit. Steffen T. Ubl and Daniel Günther were particularly involved in the design and structure of the article. Steffen T. Ubl, Thomas R. Pfeiffer, Daniel Günther, Julius M. Wehrmann, Bertil Bouillon and Alexander Bosse critically revised the article. Previous versions of the manuscript were commented by all authors. All authors have read and approved the manuscript.

## CONFLICT OF INTEREST STATEMENT

The authors declare no conflicts of interest.

## ETHICS STATEMENT

The study was approved by the Institutional Review Board and ethics committee of the University of Tuebingen (ethics proposal number 684/2019BO2).

## Data Availability

The datasets generated and analysed during the current study are available from the corresponding author on request. However, to protect patient privacy and comply with ethical regulations, any personally identifiable information has been excluded and will not be shared.
